# BETASCAN: Probable β-amyloids Identified by Pairwise Probabilistic Analysis

**DOI:** 10.1371/journal.pcbi.1000333

**Published:** 2009-03-27

**Authors:** Allen W. Bryan, Matthew Menke, Lenore J. Cowen, Susan L. Lindquist, Bonnie Berger

**Affiliations:** 1 Harvard/MIT Division of Health Science and Technology, Bioinformatics and Integrative Genomics, Cambridge, Massachusetts, United States of America; 2 Whitehead Institute for Biomedical Research, Cambridge, Massachusetts, United States of America; 3 MIT Computer Science and Artificial Intelligence Laboratory, The Stata Center, Cambridge, Massachusetts, United States of America; 4 Department of Computer Science, Tufts University, Medford, Massachusetts, United States of America; 5 Howard Hughes Medical Institute, Chevy Chase, Maryland, United States of America; 6 Department of Applied Mathematics, Massachusetts Institute of Technology, Cambridge, Massachusetts, United States of America; Fox Chase Cancer Center, United States of America

## Abstract

Amyloids and prion proteins are clinically and biologically important β-structures, whose supersecondary structures are difficult to determine by standard experimental or computational means. In addition, significant conformational heterogeneity is known or suspected to exist in many amyloid fibrils. Recent work has indicated the utility of pairwise probabilistic statistics in β-structure prediction. We develop here a new strategy for β-structure prediction, emphasizing the determination of β-strands and pairs of β-strands as fundamental units of β-structure. Our program, BETASCAN, calculates likelihood scores for potential β-strands and strand-pairs based on correlations observed in parallel β-sheets. The program then determines the strands and pairs with the greatest local likelihood for all of the sequence's potential β-structures. BETASCAN suggests multiple alternate folding patterns and assigns relative *a priori* probabilities based solely on amino acid sequence, probability tables, and pre-chosen parameters. The algorithm compares favorably with the results of previous algorithms (BETAPRO, PASTA, SALSA, TANGO, and Zyggregator) in β-structure prediction and amyloid propensity prediction. Accurate prediction is demonstrated for experimentally determined amyloid β-structures, for a set of known β-aggregates, and for the parallel β-strands of β-helices, amyloid-like globular proteins. BETASCAN is able both to detect β-strands with higher sensitivity and to detect the edges of β-strands in a richly β-like sequence. For two proteins (Aβ and Het-s), there exist multiple sets of experimental data implying contradictory structures; BETASCAN is able to detect each competing structure as a potential structure variant. The ability to correlate multiple alternate β-structures to experiment opens the possibility of computational investigation of prion strains and structural heterogeneity of amyloid. BETASCAN is publicly accessible on the Web at http://betascan.csail.mit.edu.

## Author Summary

Amyloid is a highly ordered form of protein aggregation that a wide variety of proteins can form. While the earliest discovered amyloids were associated with systemic and neurodegenerative diseases, recent findings indicate amyloids may have myriad roles and functions ranging from learning and memory, to yeast epigenetics, to biofilm and melanin production. In this study, we expand the range and flexibility of our ability to understand how amyloid properties arise from their polypeptide sequence. By taking advantage of the intrinsic properties of a characteristic amyloid structure—parallel β-strands—and data from available protein structures, we construct and test an algorithm to predict the probability that particular portions of a protein will form amyloid. Our method has the advantage of more accurate detection of the edges of such zones, as well as the ability to consider and evaluate the likelihood of multiple folding patterns.

## Introduction

“Amyloid” is a term used to describe a particular type of protein structure that can be adopted by a very wide variety of proteins with completely unrelated primary amino acid sequences [1],[2]. It is a form of protein aggregation, but of a distinct and highly ordered type. It has recently been realized that, given the right conditions, a great many, perhaps most, proteins have the potential to form amyloids. This appears to be due to intrinsic properties of the peptide backbone, a finding of great importance for understanding the evolution of protein folds. A much smaller fraction of proteins, and protein fragments, assemble into amyloid under normal physiological conditions, and these are of great interest in diverse aspects of biology and medicine[3].

Many amyloids first came to our attention because they were associated with a wide variety of diseases, from systemic amyloidoses to neurodegenerative diseases such as Alzheimer's[4]. It had initially been assumed, therefore, that amyloids were toxic species. This indeed may be the case in peripheral amyloidoses, where the massive accumulation of amyloid fibers may physically disrupt normal tissue function[5]. Increasingly, however, evidence suggests that the formation of amyloids may more commonly be a protective mechanism which, especially in the case of the neurodegenerative amyloidoses, acts as to sequester misfolded polypeptides that would otherwise dwell in more toxic, and more highly interactive, oligomeric species. It has also recently been realized that amyloids serve important biological functions in a number of different situations. For example, in melanocytes, amyloid fibers formed by Pmel17 play a role in the production of melanin[6], and in bacteria extracellular amyloids are a key feature of the biofilms that are so difficult to eradicate in various infectious processes[7]. In fungi, a special class of self-templating amyloids serve as elements of inheritance: these bi-stable proteins can persist as soluble or amyloid species and the change in function that occurs when with the switch to the amyloid form, is passed from generation to generation as mother cells faithfully pass amyloid (prion) templates through the cytoplasm to their daughter cells[8],[9]. Such self-perpetuating prion-like switches in state also appear to play a role in neuronal learning and memory, by maintaining a translation factor involved in the maintenance of synapses in an active and highly localized state[10]. There is, therefore, great interest in deciphering the structures that underlie amyloid states.

Several methods have established that amyloids are generally rich in β-strands aligned perpendicular to the long axis of the fibril [11],[12],[13],[14],[15]. Beyond this, frustratingly little is known about their structure. Crystallization for X-ray structure determination has proven impossible except for extremely short segments [16],[17]. Notably, the importance of interactions between side-chains in these structures establishes that a detailed understanding of such interactions will be necessary to comprehend the physical and biological properties of other amyloids. The insolubility of amyloids has also precluded NMR-based structural determination until very recently, when solid-state nuclear magnetic resonance (ssNMR) studies have yielded partial, specifically parallel β-structures in a few specific cases [18],[19],[20],[21],[22]. Due to the scarcity of direct evidence, the nature of amyloid and prion supersecondary structures and their relation to sequence have been highly contentious topics [16],[23],[24]. The debate has been complicated by the morphological heterogeneity of amyloid structures suggested by EM imagery [25],[26] and the demonstration of prion ‘strains’ or ‘variants’ with differing growth and stability phenotypes [27],[28],[29]. In the case of the yeast prion protein Sup35, such variants have been demonstrated to maintain specificity through serial passage [28] and have been correlated with differences in conformation [30]. These results underscore the need to consider alternate supersecondary structures for amyloid and prion strands.

Given the difficulty of direct observation of supersecondary structure, computational modeling of amyloid folding has been attempted. Unfortunately, barriers exist to the effective application of sequence-based computational analysis. Several homologous prion-forming domains, while functionally conserved over evolutionary time, have sequence identities of under 25%, with sufficient additional rearrangement as to preclude multiple sequence alignment via standard algorithms such as CLUSTAL [31]. Analysis of amyloidogenic proteins has not revealed overall commonalities of sequence, except in individual residue frequencies [32],[33],[34] and a tendency for imperfect repeats to appear [35],[36]. Secondary structure prediction algorithms [37],[38] identify many amyloid- and prion-forming domains as random coil without structure. The amyloid-forming domains of these sequences are removed by the low-complexity filters of local sequence alignment tools such as BLAST [39], rendering another family of methods ineffective.

The strong evidence for β-structure in amyloid suggests that, as an alternate means of secondary structure prediction, computational methods designed to predict globular β-structure should be assessed. BETAWRAP [40],[41] was the first program to incorporate the important long-range pairwise interactions into a computational method to predict β-structure. In doing so, BETAWRAP was the first program to predict *strand-pairs*, defined as any two β-strands connected by the hydrogen bonds of a β-sheet. The program is restricted by a template of strand lengths to predict only one sub-family of the parallel β-helices, a fold widely cited as similar to amyloid [25],[42],[43]. BETAPRO [44] is a general method that incorporates pairwise properties into a neural net to learn globular β-strands and strand-pairs.

A variety of other approaches have been implemented in the search for a reliable detector of protein aggregation. TANGO[45] utilizes a statistical mechanics approach to make secondary structure predictions, including differentiation of beta-aggregation from beta-sheets. The TANGO algorithm presumes that all residues of an aggregate will be hydrophobically buried. Zyggregator [46] models aggregation propensity per residue as a combination of four factors intrinsic to a sequence: charge, hydrophobicity, secondary structure propensity, and the “pattern” of alternating hydrophobic and hydrophilic residues. Zyggregator derives its statistical basis from a study of effects of mutation on aggregation [47] and calculates its scores based on a sliding window of 21 residues. SALSA [48] uses a sliding window to sum the cumulative Chou-Fasman parameter score, then selects the 400 best scores and sums each residue's contribution. Finally, PASTA [49],[50] calculates singleton and pairwise propensities for individual residues and residue-pairs by calculating a weighted average of the contribution of that residue or pair to β-strand formation. The pairwise scores, in turn, were calculated according to a Boltzmann energy function derived from the adjacencies in a database of 500 annotated structures.

We introduce a program, BETASCAN, to predict prions and amyloids as well as other forms of parallel β-structure. Like PASTA, BETASCAN relies on calculation of strand propensities. However, BETASCAN makes use of a novel hill-climbing algorithm to find the most preferred β-strands and strand-pairs. Our hypothesis is that BETASCAN will be able to determine the location and length of the β-strands present in the amyloid and prion protein sequences. Coupled with a more statistically robust method to estimate pair propensities and the consideration of the amphipathic environment of amyloid β-sheets, the hill-climbing method leads to favorably comparable performance by BETASCAN compared to previous methods, as determined by existing experimental data.

## Results

BETASCAN was designed, in principle, to predict parallel β-structure in all cases where the two surfaces of the β-sheet have significant environmental differences. Our strongest subset of interest within this area of competence was the set of prion and amyloid proteins. We therefore tested BETASCAN on five amyloids with known structures and a set of aggregating proteins. In order to verify the accuracy of BETASCAN predictions, we ran BETASCAN on a non-redundant set of crystallized parallel β-helix proteins. This set of structures provided the closest analogue to prion and amyloid proteins with detailed crystal structures available.

### Test sets

In addition to testing BETASCAN and competing programs on amyloids, we also test them on their ability to detect β-strands in a superfamily of parallel β-folds with solved crystal structures, namely the parallel β-helices. Here we test BETAWRAPPRO (the improved version on the BETAWRAP algorithm specifically designed for predicting β-helices), BETAPRO (the neural network for predicting β-strands), PASTA, SALSA, TANGO, Zyggregator, and our program BETASCAN for correct detection of β-strands in a β-helix data set. In addition, we compare our program's predictions to those of the other algorithms in light of the known experimental structural evidence for amyloid proteins.

### Available verification data

X-ray crystallography results were available from 34 independent, non-redundant structures of β-helix sequences excluded from the pairwise and singleton probability tables (Table S1). In addition, deuterium-exchange solid-state NMR β-sheet detection results were available for amyloid A-β [18],[19], the *Podospora* Het-s prion [20],[21], portions of α-synuclein [51], and the PHF43 fragment of the tau protein [52]. A theoretical model of amylin/islet amyloid polypeptide [53] was also used in the verification of PASTA, and its analysis was included as well. An additional structure of A-β was considered [54], but was too low in resolution to determine lengths of component beta-strands.

### Output formats

**Figure 1:**
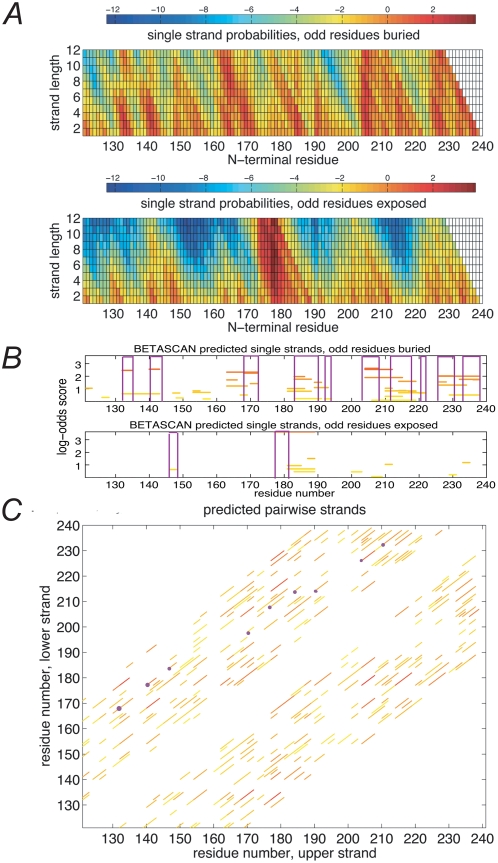
Sample output of the BETASCAN algorithm. The results for the β-helix domain of pectate lyase C are shown. (A) heat-map of all β-strand probabilities. The horizontal axis indicates the N-terminal residue of potential β-strands, while the vertical axis indicates strand length. The upper and lower boxes display results for the two orientations of the strand. Colors indicate propensity of strand formation. Red indicates above-background probability, while blue indicates below-background probability. (B) predicted most likely β-strands based on single strand probabilities. BETASCAN predictions are marked as horizontal lines, shading from red (maximum predicted score) to yellow (zero score, i.e., probability equal to background). The horizontal axis indicates the N-terminal residue of potential β-strands, while the vertical axis indicates the log-odds propensity. Overlapping strands represent alternate folding patterns with indicated likelihoods. Purple brackets indicate experimentally determined β-strands as derived from the PDB structure. (C) predicted most likely strand-pairs based on pairwise probabilities. Purple dots indicate the N-terminii of experimentally determined strand-pairs as derived from the PDB structure.

As an example of typical BETASCAN visual outputs, Figure 1 presents sample outputs from BETASCAN for the β-helix domain of *Erwinia crysanthemi* pectate lyase C. The heat-map at top (Figure 1a) depicts the assignment of a likelihood score to each point on a lattice of possible β-strands. In these graphs, the starting point of a putative β-strand is indicated horizontally, and the length of such a β-strand increases vertically. Organized in this fashion, a likely β-strand appears as a triangular signal of high probability against a low-probability background; the strand location and length may be read at the triangle's apex. The residues on the strands may face in one of two directions (starting inward or outward, relative to fibril core); therefore, two graphs are presented to depict the effects of residue orientation.

The strands with local maximal likelihood were calculated for output as described in Materials and Methods; Figure 1b offers a concise version of the results. Here, the potential β-strand lengths and locations are depicted horizontally; the vertical axis indicates the score for each potential strand. An analogous procedure was then executed for all strand-pairs, resulting in the set of local maximum likelihood strand-pairs depicted in Figure 1c. As in Figure 1b, strand-pairs lengths and locations are depicted, with the horizontal and vertical axes indicating the starting points in the sequence of the first and second strands of the pair.

Predictions of specific β-strands and strand-pairs may now be made directly from the sets of local maximal likelihood structures. For instance, the marked strands and strand-pairs in Figures 1b and 1c are the set of non-overlapping structures with the highest score. These correspond well to the β-strands and strand-pairs observed in the PDB-deposited crystal structure (purple bars in Figures 1b and 1c). Confidence in the prediction for any strand, strand-pair, or subsequence thereof may be inferred from the additional predictions for the location.

### Verification from crystal structures of β-helices

β-helices have been widely noted as the closest globular protein analogue of amyloid and prion structures [25],[42],[43],[55]. Because of this similarity, the β-helices were removed from consideration during the computation of the probabilistic database. Therefore, these structures formed a useful test set to evaluate the accuracy of the BETASCAN algorithm in β-strand detection. The BETASCAN results for the non-orthologous β-helices (listed in Table S1) were compared to the STRIDE analysis of β-strands in crystal structures. Statistics were collected on the accuracy of predictions by strand and by residue, and on the accuracy of left- and right-edge locations.

**Figure 2 pcbi-1000333-g002:**
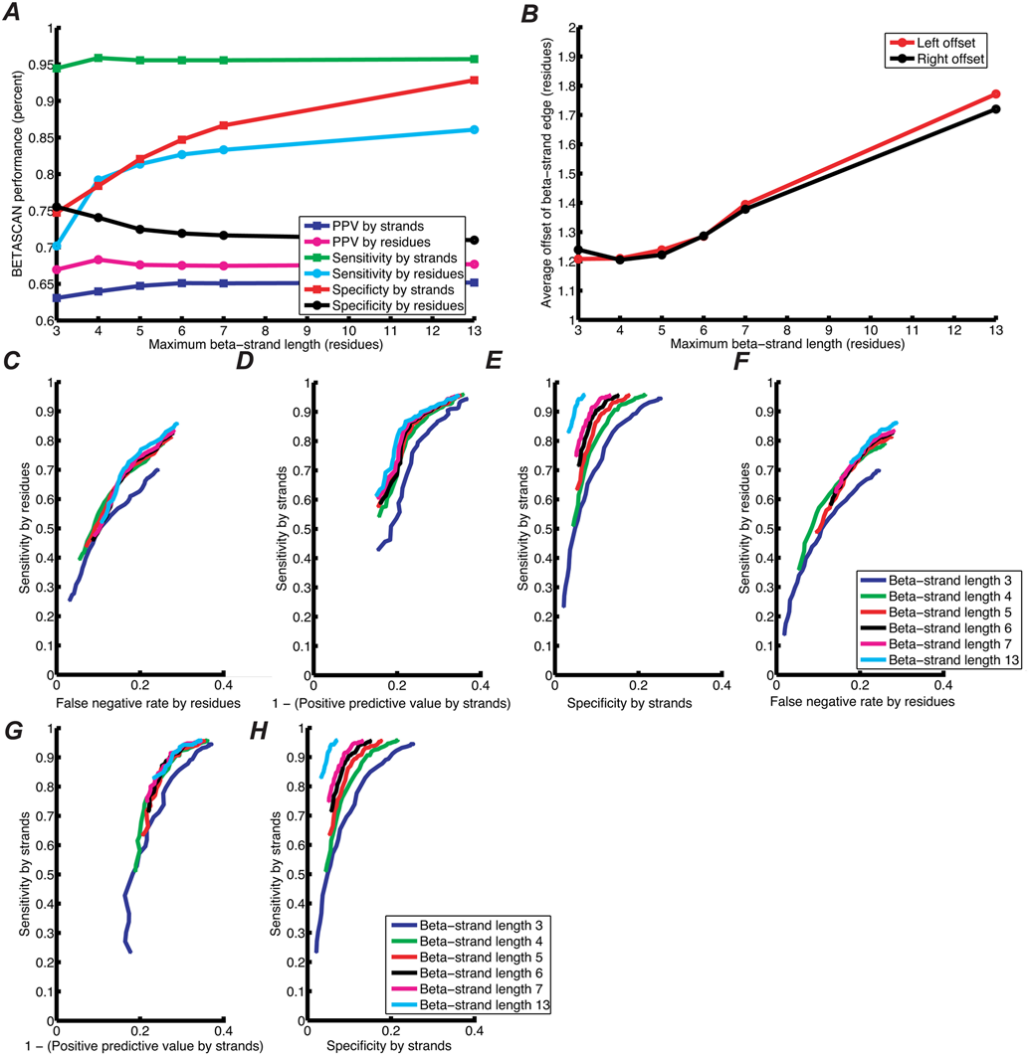
Statistics on BETASCAN accuracy in the set of β-helices. (A) effect of maximum β-strand length on sensitivity and positive predictive value of BETASCAN, measured by strand and by residue. (B) effect of maximum β-strand length on average absolute value difference in predicted and crystal-structure observed β-strand edges. (C–E) effectiveness of BETASCAN singleton scores in β-structure prediction; (C) ROC curve calculated residue-by-residue; (D) graph of sensitivity against (1-PPV) calculated strand-by-strand; (E) ROC curve calculated strand-by-strand. (F–H) effectiveness of BETASCAN pairwise scores in β-structure prediction; (F) ROC curve calculated residue-by-residue; (G) graph of sensitivity against (1-PPV) calculated strand-by-strand; (H) ROC curve calculated strand-by-strand.

The accuracy of BETASCAN, counted by strand and by residue, is depicted in Figure 2a. Examined strand-by-strand, BETASCAN had an effective sensitivity of 94–96% to correct strands. In addition, as long as the maximum β-strand length is equal or greater to the average β-strand length, BETASCAN achieved 80% or greater sensitivity, measured residue-by-residue, for this data set. As shown in Figure 2b, the error in the predicting the left and right edges of each strand was between one and two residues each. The error in edge localization was minimized when the maximum β-strand length was closest to the average β-strand length, and increased considerably when longer strands were considered. The residue-by-residue sensitivity is thus reflective of the error in edge localization.

Our hypothesis that pairwise probabilities reflect the occurrence of β-strands in structures necessarily implied that the scores discriminate between β-forming subsequences and sequences that form loops or other structures. Figures 2c, 2d, and 2e describe the sensitivity and specificity or positive predictive value of BETASCAN scores for residues and for strands. Sensitivity and specificity, measured by residue, were markedly reduced if strands of sufficient length are not considered, while additional strand length had little or no effect. However, strand-by-strand sensitivity, specificity, and PPV were slightly improved. For all lengths and scores examined, negative predictive value (NPV) was 95% or higher.

While portions of a loop may be found in β-conformation without the distinctive hydrogen bonds of a β-sheet, the majority of β-strands are found in β-sheets. Therefore, the scores of the predicted pairwise contacts may indicate whether a given postulated β-strand is present in native or amyloid structures. With this hypothesis in mind, a filter was devised to exclude strands without significant associated pairwise contacts from the BETASCAN single-strand maxima results. A strand was considered to have poor pairwise contacts if the summed scores of pairwise contacts with the strand as the first element was less than some threshold value. Figures 2f–2h reveal the effect of increasingly filtering pairwise-poor strands on sensitivity, specificity, and strand-by-strand PPV for the β-helix test set. The best results for this set, as determined by the receiver-operating characteristic method, were at a pairwise filter threshold score of 17. For strands with summed pairwise scores above this value, 91–94% sensitivity and 84–94% specificity was observed, with the longest allowed strand lengths yielding the best statistics. Measured residue by residue, 72–83% sensitivity and 74–81% specificity were achieved, with the best statistics observed at a maximum length of four residues. PPVs achieved were 68–70% for strands and 70–73% for residues.

### Comparison to BETAWRAPPRO results

Comparisons were also made between the BETASCAN β-helix results and the highest-scoring predictions of BETAWRAPPRO, the latest BETAWRAP algorithm [41]. Since the BETAWRAPPRO algorithm incorporates structural information specific to β-helices, comparison of BETASCAN and BETAWRAPPRO indicates the relative utility of structure-specific knowledge.

BETAWRAPPRO predicted 276 strands in its top results for each of the β-helices studied. When compared to the 631 β-strands in the crystal structures, 189 were found to correspond, for a sensitivity of 30% and a positive predictive value of 68.4%. Of these 189 strands, 183 strands were considered matched by BETASCAN under the same conditions used for matching in the BETASCAN analysis above. These results were unchanged by changes in maximum β-strand length. Thus, BETASCAN effectively reproduces the correct results of BETAWRAPPRO without structure-specific knowledge. While markedly increasing sensitivity to β-strands, especially to those outside the canonical β-strand pattern, BETASCAN also maintains the positive predictive value achieved by BETAWRAPPRO.

### Verification from solid-state NMR analyses of amyloids, and comparison with PASTA and SALSA

Solid-state NMR analysis was used by Petkova et al. [18], Luehrs et al. [19], Ritter et al. [20], and Wasmer et al. [21] to determine strand-pair contacts in A-β 1–42 and the *Podospora* Het-s prion. Briefly, ^1^H-NMR signals were taken before and after one week of immersion in D_2_O. Deuterium exchange occurred in all locations except where retarded by the energy wells of hydrogen bonds, allowing the identification of residues taking part in β-sheet hydrogen bonding.

**Figure 3:**
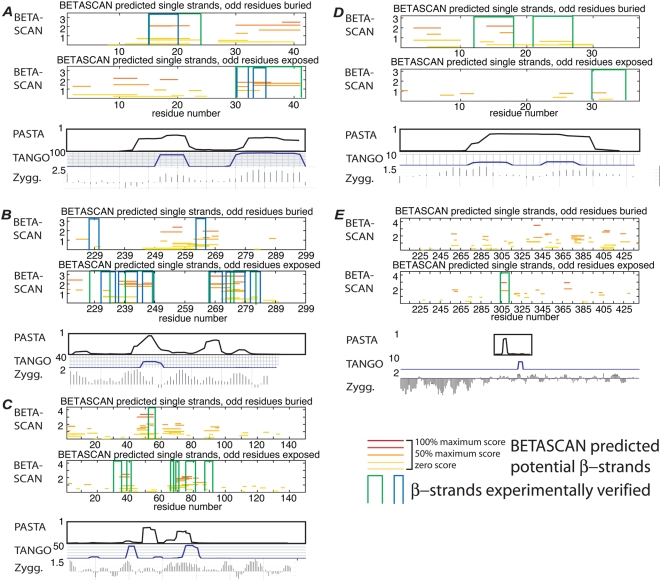
BETASCAN output for amyloid and prion proteins with experimentally determined β-structures. Green vertical brackets indicate experimentally derived locations of β-strands; blue brackets indicate locations determined by a separate method. In the same manner as Figure 1b, BETASCAN predictions are marked as horizontal lines, shading from red (maximum predicted score) to yellow (zero score, i.e., probability equal to background). Overlapping lines indicate alternate folding patterns for the β-strands, with indicated probability. Two graphs are included to display the results for each orientation of the strand. For purposes of comparison, the set of highest-scoring non-overlapping strands in the BETASCAN single-strand prediction was taken as the predicted structure. Corresponding outputs of PASTA [49],[50], TANGO [45], and Zyggregator [46] are displayed below the BETASCAN results. Refer to Table 1 for a summary of the correspondences of these predictions. (A) amyloid-β structure as determined by Luehrs et al. [18] (green) and Petkova et al. [19] (blue); (B) het-S structure as determined by Ritter et al. [20] (green) and Wasmer et al. [21] (blue); (C) α-synuclein structure as determined by Heise et al. [51]; (D) amylin structure as determined by Kajava et al. [53]; (E) tau protein fragment PHF43 structure as determined by von Bargen et al. [52].

The structure of A-β 1–42 (Figure 3a) under differing conditions was determined independently by Petkova et al. [18] and Luehrs et al. [19]. As determined by Luehrs, the structure included two β-strands formed at residues 15–24 and 30–42, each forming in-register interchain strand-pairs. The structure as determined by Petkova included a strand from 10–14 and a region from 30–35 that was ambiguously determined as one or two strands. Predictions by PASTA and SALSA suggested β-structure in the regions 10–22 and 29–42 without elaboration. The BETASCAN algorithm, as its top specific prediction, produced β-strands at residues 9–13, 15–22, and 30–42.

The heterokaryon compatibility prion Het-s from *Podospora anserina* [12] was found by Ritter et al. [20] to form four β-strands, with one β-sheet composed of alternating copies of β-strands 1 and 3, and another β-sheet similarly composed by strands 2 and 4 (Figure 3b). The results of Wasmer et al. [21] indicated breaks in each of these four β-strands, thus predicting a total of eight closely spaced strands. In addition, the new results indicated a reversal of orientation at the breaks in strands 1 and 3. PASTA predicted two strands and the possibility of a third, corresponding to Ritter's strands 2, 3, and 4. The BETASCAN algorithm strongly predicted Ritter's strands 2, 3, and 4 at their full length. While BETASCAN's prediction matched only the C-terminal half of Ritter's strand 1, it matched both strands 1a and 1b of the Wasmer model at lower probability. Wasmer strands 2a, 2b, 3a, 3b, and 4a were all indicated at high probability, and Wasmer strand 4b at the same probability as strand 1a. Each of the strand-pairs observed by Ritter and by Wasmer was found in the strand-pair set predicted by BETASCAN, although the signal was not clearly distinguishable from other potential pairings.

α-synuclein has been analyzed by Heise et al. [51] to contain a total of seven strands. The two highest-scoring, the third and sixth strands, were detected by both PASTA and BETASCAN with high accuracy (Figure 3c). SALSA predicted the first two, with a large and vague prediction of amyloid propensity covering the remaining strands. While some predictions were low-scoring, only BETASCAN indicated the possibility of the seven strands detected by experiment.

Amylin, also known as islet amyloid polypeptide (IAPP), was modeled by Kajava et al. [53] to be an in-register amyloid composed of three strands. PASTA predicted an amyloidogenic region from 15–32. BETASCAN results (Figure 3d) suggested two of the three strands predicted by Kajava, part of the third strand (30–33), and an additional strand at residues 3–7. This potential strand may be related to the intrachain cysteine bond between residues 3 and 8.

Aggregation in the tau protein centers on the repeat domain, which takes the conformation of random coil in the native state [52]. Interest has more specifically centered on the protease-resistant PHF43 sequence, though other regions of the protein product have been suggested to play roles [56]. Trovato and colleagues only analyzed the PHF43 domain itself, verifying the importance of the hexapetpide VQIVYK at residues 306–311. Here, the region between residues 205 and 441 is analyzed. A more extensive run of the PASTA algorithm finds strands at 258 and 338. SALSA weakly detects strands at about 235, as well as at 390 and 410. The more expansive BETASCAN analysis presented in Figure 3e underscores the importance of residues 306–311, as it is the most likely β-strand to form in the entire tau protein; it also detects strands at 255, 338, and 390–410.

### Comparison across multiple prediction programs

A synopsis comparing the predictions of BETASCAN to those of PASTA (as provided in [50]), SALSA (as provided in [48]), TANGO [45], Zyggegator [46], and BETAPRO[44] is presented in Table 1. As a control, the results of JPRED [37] and PSIPRED[57] are included to represent traditional secondary structure prediction. Because of the difficulty in translating one program's scores to another's, predictions were indicated as ‘strong’ or ‘weak’ depending on relative internal scoring and extent of prediction. For IAPP, Het-s, and α-synuclein, our program BETASCAN was the only program to detect the correct number of strands. All algorithms successfully predicted the strands of A-β, although some did not detect all of strand 2. All algorithms were also able to detect the strongest strands for the tau protein, except that BETAPRO and TANGO did not detect the first strand near residue 235. BETAPRO tended to miss strands, while PASTA, SALSA, and Zyggegator had difficulty separating strands. TANGO tended to miss strands at the edges of β-rich regions.

**Table 1:** Comparison of BETAWRAP results to previous algorithms.

Protein	A-β		Het-s				α-synuclein							IAPP			Tau			
Strand	1	2	1	2	3	4	1	2	3	4	5	6	7	1	2	3	1	2	3	4
**BETASCAN**	**S**	**S**	**w**	**S**	**S**	**w**	**w**	**S**	**S**	**w**	**S**	**S**	**S**	**S**	**w**	**w**	**w**	**S**	**S**	**S**
BETAPRO	S	w	n	n	n	S	n	S	n	w	n	n	w	n	n	n	n	S	S	w
TANGO	S	S	n	S	n	n	n	S	w	w	w	S	n	w	w	n	n	S	n	n
Zyggregator	S	S	(+)		(+)		n	S	w	(+)			w	S	(+)		S	w	S	n
PASTA	S	S	n	S	w	n	n	w	S	w	w	S	n	(+)		n	S	S	S	w
SALSA	S	S	x	x	x	x	n	S	S	(+)				x	x	x	n	S	w	w
PSIPRED	S	S	n	w	w	w	n	S	w	n	n	n	n	n	n	n	n	n	S	n
JPRED	S	S	w	S	w	w	n	S	S	w	S	S	w	n	w	n	n	w	S	w

Letters indicate strength of prediction: S, strong (complete prediction); w, weak (missing >30% of length or <50% confidence); (+), prediction without strand boundaries; n, not predicted; x, data not available.

### Analysis of aggregating sequences

**Figure 4:**
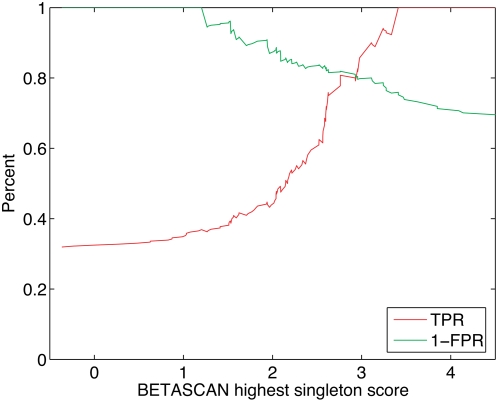
Sensitivtiy/specificity curve of BETASCAN highest singleton result for 120 non-redundant sequences experimentally observed for aggregation potential, collated by [45] and clustered by CD-HIT [58].

We also used BETASCAN to analyze a larger database of sequences derived from proteins observed to aggregate in experimental settings, and the results compared favorably to those of PASTA (see Figure 4). The data set previously used for analysis by [45],[49] was considered as a benchmark. However, the redundant content of the set was found to cause a loss of robustness, as determined by an analysis involving the removal of one or two clusters' redundant sequences. (see Table S2). Therefore, a non-redundant version was calculated by CD-HIT [58] with 40% sequence similarity cutoff, and the resulting 120 sequences (see Table S3) were analyzed by BETASCAN. The specificity and sensitivity curves for the top beta-strand score of each sequence intersect at 81%, which compares favorably to previously reported PASTA results [49].

## Discussion

We have introduced the program BETASCAN and showed its improved performance over previous methods for identifying β-strands in parallel β-structures, most importantly amyloid structures. The BETASCAN approach depends upon the idea that, while all sequences display some tendency towards the β conformation, sequence details determine the relative likelihood of β-strand and strand-pair formation at all scales. Thus, sequence has a broad effect not only on secondary structure, but also on the supersecondary structural assembly of β-strands into a β-sheet. This concept is the driving force of both the scoring and maxima-finding algorithms in BETASCAN. The score is designed to allow unbiased comparisons between β-strands differing not only in sequence, as in BETAWRAP, but also by length and orientation. Correspondingly, the maxima-finding algorithm uses these comparisons to explore strand and pair space for locally optimal β-strands and strand-pairs.

In addition, BETASCAN may owe some of its strong performance, compared to PASTA, to its ability to distinguish strands of different lengths in relation to their rate of occurrence in nature. PASTA scores are generated for residues and residue pairs based upon the weighted-sum scores of every potential β-strand that could be formed using that residue or residue pair. In contrast, the emphasis in BETASCAN is placed upon finding specific high-scoring strands. Any region with a high PASTA score will also contain high-scoring BETASCAN predictions, which supply additional information about where strands are likely to begin, end, and pair. The concentration on the strand as the fundamental unit of β-structure also improves residue-by-residue detection of β-structure.

BETASCAN is highly sensitive for potential strands and excellent at determining when sequences will not contribute to β- and amyloid structure (high negative predictive value). However, the effort to identify all potential β-structure variants can cause significant over-prediction of β-structure, as Figures 1–3 all reflect. While the highest-scoring strands consistently reflect real structures, only some of the lower-scoring strands are found in experimental data. The low-scoring strand at residue 146 in the 2PEC structure (Figure 1b) is an example of a low-scoring strand extant in crystal structure. By synthesizing the singleton and pairwise maxima results of BETASCAN, a better predictive capacity is achieved. The optional pairwise-based filter demonstrated in Figures 2f–h can identify structural strands with better performance than exclusion by low score alone, and retains low-scoring strands that readily form strand-pairs. Additional factors, discernable by experimental data or by more astute analysis, may be used as additional specificity filters to distinguish which *potential* β-strands are contributors to either amyloid or native structures. However, the knowledge that amyloid structures include multiple ‘strains’, may be heterogeneous even within a single fibril, and frequently include β-strands not found in the native fold of the parent protein, argues for the inclusion of hypothetical β-strands in analysis until excluded by evidence.

Most interestingly, BETASCAN is capable of revealing details and variants of protein structure previously inaccessible to computational methods. For instance, two solid-state NMR studies of A-β protein [18],[19] produced conflicting results in the region between residues 30 and 42. However, each result is reflected in the BETASCAN results (Figure 3a), where both short strands corresponding to the Petkova results and long strands corresponding to the Luehrs results are high-scoring maxima. Likewise, two solid-state NMR studies of Het-s [20],[21] were differentiated by the presence or absence of interruptions in the β-strands. Both the elongated and truncated versions of these strands were isolated by the maxima-finding subroutine of BETASCAN. Thus, BETASCAN can distinguish the local attractor states that the two pairs of experimental samples occupied, opening the possibility of understanding the influence of environmental conditions and/or folding kinetics on “prion strains” and other amyloid folding variations.

BETSACAN forms part of a synergistic strategy for the evaluation of all-β structure. Additional β-strand specificity may be found using experimental contextual clues, such as discernment of physical attributes, specific links between residues such as cysteine bonds and side-chain ladders, and constraints on the conformational space of the amyloid. The variants indicated by BETSACAN may also be distinguished *in vitro* or *in* vivo by additional exterior factors, such as pH, osmolarity, and the presence of seeding factors or chaperone proteins. By distinguishing folding variants and providing specific location and likelihood data, BETASCAN thus boosts to the efficacy of both experimental and computational efforts to understand the parallel β-sheets of amyloids and prions.

## Materials and Methods

### Algorithmic strategy

BETASCAN calculates likely β-strands and strand-pairs for an input sequence presumed to contain parallel β-structure. Every contiguous subsequence of length 2 up to *k* is initially considered as a possible parallel β-strand (*k* defaults to 13, the length of the longest parallel β-strand in our source database.) For each pair of possible strands, a score is determined corresponding to a prediction of how likely their pairing would be (see **Strand state propensity** and **Pair state propensity** below). This probability is based on the observed preferences for each pair of residues in the strands to be hydrogen-bonded (see **Probability tables** below). Maxima-finding algorithms, also known as “hill-climbing” algorithms, are then used to detect all local maxima of formation propensity across strand-pair space (see **Maxima finding** below). The outputs of BETASCAN are score-ordered lists of all locally optimal strands and strand-pairings.

Note that BETASCAN can return strands and/or strand-pairs that inconsistently overlap in the local-optimum list. These results reflect the potential, under differing conditions, for alternate β-strand folding patterns.

### Probability tables

**Figure 5:**
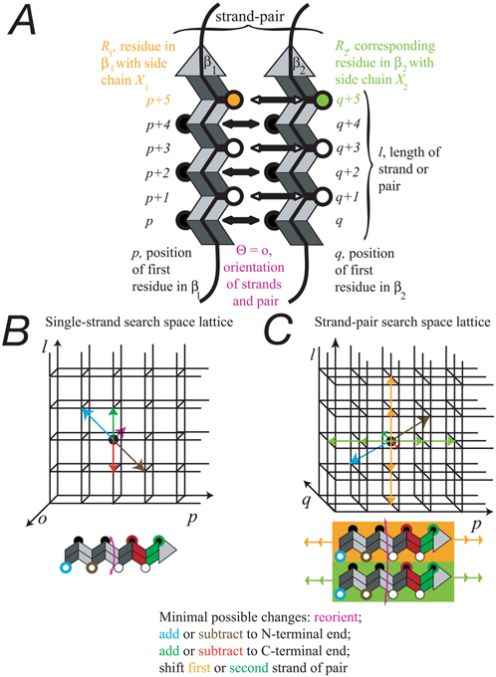
Relationships between physical features of β-sheet components and the definition of the computational search spaces. (A) variable definitions as used in *Materials and Methods*. The two vertical beta-strands form a single strand-pair, with odd residues labeled in white and even residues in black. The strands share the same orientation *o* and extend from *p* to *p+l* and from *q* to *q+l*. (B) structure of the lattice of the β-strand search space defined by the variables *p* (location), *l* (length), and *o* (orientation). Changes in the parameters of a β-strand are physically possible in a single step only along the paths marked by arrows. The arrowheads therefore define the relative locations queried by the maxima-finding algorithm at each point. (C) structure of the lattice of the strand-pair search space defined by the variables *p* (first strand location), *q* (second strand location), *l* (length) and *o* (orientation, not shown). In addition to the physically possible changes in B, shifts of one or two residues in the relative strand positions are possible. Arrowheads indicate the relative locations queried by the strand-pair maxima-finding algorithm for each point.

Pairwise probability tables to capture the preference for each pair of amino acids to be hydrogen-bonded in a β-sheet was estimated using a method similar to McDonnell *et al.* [41] Briefly, the non-redundant structures of the Protein Data Bank [59] as of June 8, 2004, were filtered to remove the set of structures in Table S1. These structures, including all three-stranded right-handed β-helices, were removed for two reasons. First, their similarity to known and theorized amyloid structures was considered a potential source of bias. Second, their removal allowed their use as a control test set (see **Test set construction** below). The STRIDE algorithm [60] was used to on the remaining structures to find all amphipathic β-sheets, namely β-sheets with solubility differences between its two faces. The frequencies of occurrence of hydrogen-bonded pairs *(X_1_,X_2_,θ)* were tabulated, where the orientation *θ* distinguished β-sheet faces with lesser (zero) or greater (one) solubility. The frequencies were normalized to sum to 1, generating the 20×20×2 pairwise statistical table *W*. (Symbols identify structures as indicated in Figure 5a.)

*V*, the 1×20×2 singleton probability table, represents the propensity of a side-chain *X_1_* to be present in an amphipathic β-strand. *V* was calculated by summing the pairwise probability tables across rows.

Background probability tables were generated by counting single amino acid frequencies across all protein sequences (not only β-structures). Background probability pairwise tables were formed by squaring the singleton frequencies, corresponding to an independence assumption for the null hypothesis. The default table *C_allproteins_* is derived from the release 50.4 (July, 2006) of the SWISS-PROT database. Prion and amyloid sequences derived from genomes of yeast species with amino acid distributions potentially biased by sparse GC content, as determined by whole-genome phylogenetic analysis, were analyzed using a table *C_allyeasts_* derived from the genome of *Saccharomyces cervisiae* as of July, 2006.

### Strand state propensity

Figure 5a serves as a visual reference for the following formulas. For a possible beta-strand starting at position *p* with length *l* and orientation *o*, the propensity *h(s, p, l, o)* of formation for a strand state *(p, l, o)* forming from a polypeptide sequence *s* is calculated as the ratio of the propensity *f(s, p, l, o)* of the strand sequence to form a β-sheet and the propensity *g(s, p, l)* of the strand sequence to occur randomly. The background propensity *g* is calculated as the product of the occurrence rates *c* of each residue in the possible strand, derived from the background table *C*. (The table *C_allproteins_*, as derived above, is the default for *C*.) The strand propensity *f* calculation similarly begins by multiplying each residue's frequency *v* in the singleton probability table *V* (as calculated above) for the orientation *o*. The calculation of *f* also includes dividing by a length correction term to model the effect of length on the formation of a β-strand. The length correction term is included to enable comparison of strands with different lengths on an equal basis, a requirement for the maxima-finding subroutine (see **Maxima finding** below). The form of the correction was chosen to reflect the observed histogram of parallel strand-pair lengths in the PDB [61]. The best-fit curve of this independently derived data was found to be a Poisson distribution with parameters (*l* - 1, 3.15). A potential explanation for the Poisson distribution is the modeling of each residue's addition to the β-strand as a Poisson process.

Including the correction term, the propensity of formation is therefore
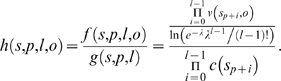



### Pair state propensity

Given a second strand starting at position *q*, the propensity *k(s, p, q, l, o)* of formation for a parallel pair state (*p*, *q*, *l*, *o*) from one or more copies of a polypeptide sequence *s* is calculated in a fashion similar to that above. (See Figure 5a for a complete visualization of the structure under consideration.) The calculation of *k* incorporates the single-strand propensity *h(s, p, l, o)* of the first strand, the composition propensity *g(s, q, l, o)* of the second strand, and the pairwise propensity *j(s, p, q, l, o)* of the two strands' adjacency. The pairwise propensity *j*, is calculated from the pairwise propensity table *W* by multiplying terms *w* for each pair of residues and dividing by the length-correction term. The inclusion of *h* in the calculation of *k* is necessitated by the form of *W*, which pre-supposes the formation of the first β-strand.
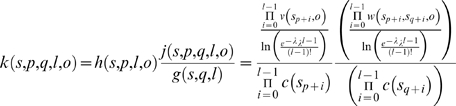



### Maxima finding

The maxima finding subroutine of BETASCAN extracts the most likely strands and strand-pairs by asking if a single change to the strand or strand-pair would result in a higher probability of formation. Not all transitions between strand states or strand-pair states are physically realizable in one step. The constraints on the strand and pair spaces may be described as lattices, with nodes corresponding to each potential strand or strand-pair and edges corresponding to the conformational changes required to form one potential strand from another. Edges may be formed by the addition or removal of residues at either end, by the reversal of strand orientation (180° rotation around the long axis of the strand), and for strand-pairs, the shearing of the strands' interactions by one or two residues. The possible transitions, and the lattices so created, are depicted in Figures 5b and 5c.

The BETASCAN method assigns a propensity to each node of these lattices, with the highest score corresponding to the most likely strand or strand-pair. A hill-climbing method, which searches each node's adjacent neighbor for a higher score, is then executed across the entirety of strand and pair space. All nodes with at least one such neighbor are removed from consideration. The remaining sets of strand states and pair states are local propensity maxima in strand and pair space. Together with their propensity scores, these sets form the output of the BETASCAN algorithm.

To allow comparisons with other prediction methods and to highlight the most relevant strands and pairs, filtering was applied. Only those strands and pairs with positive log-odds propensity scores, indicating a propensity of formation greater than random sequence, were selected. For the results in the **Comparison** sections, a consistent set of strands was chosen by repeatedly selecting the highest scoring strand that was consistent with all previously selected strands until the list of potentially consistent strands with scores more likely than random was exhausted.

### Test set construction

3-D crystal structures of the β-helices removed from the probability database (listed in Table S1) were downloaded from the Protein Data Bank [59]. The β-helix test set structures were chosen as non-redundant representatives of SCOP families, without substrates or other co-crystallized molecules. β-strands and strand-pairs were identified using STRIDE [60] as described in McDonnell et al. [41].

The all-β secondary structures of the input sequences were verified. For the β-helix sequences, 3-D X-ray crystallography was available to guarantee secondary structure details [40]. In addition, the sequences were analyzed by the secondary structure prediction program DSSP [62] to localize α-helical content.

### Comparison calculations

BETASCAN, BETAWRAPPRO, BETAPRO, and PASTA were run on the 34 β-helix structures listed in Table S1. Because β-helix strands are, on average, just over four residues in length, BETASCAN runs were executed using maximum β-strand lengths of 3, 4, 5, 6, and 7, as well as with the default length of 13. A single consistent set of predicted strands was selected from the set of all predicted strands by repeatedly selecting the strand with the highest positive score that failed to overlap either any previously selected strand or any α-helix (as observed in crystal structure by DSSP [62]. The set of predicted strands was compared to the β-strands calculated from crystal structures by the program STRIDE [60] according to the settings of McDonnell et al. [41]. The STRIDE predictions were taken as the true positive β-strands for this class.

For each real strand, if at least one predicted strand overlapped more than 50%, a match was recorded. In addition to the fraction of matching crystal and predicted strands, statistics were collected on the number of matching residues and on the predictions of β-strand ‘edges’. The N- and C-terminal ends of the crystal strand were compared, respectively, to the N- and C-terminal ends of the N- and C-most matching predicted strands. In most cases, only one predicted strand matched the crystal strand, and so the ends compared were the N- and C-terminal ends of the prediction.

To generate ROC and sensitivity/PPV curves (Figures 2c–2e), the output of BETASCAN was repeatedly analyzed with a lower-bound score cutoff, which was varied from 0 to +2 units. For the poor-pairwise-contact filter (Figures 2f–2h), ROC and sensitivity/PPV curves were generated as follows. For each strand in the BETASCAN singleton results, the scores of all strand-pairs sharing the first residue of the first pair (parameter *p*) with the predicted β-strand were summed. The β-strand was removed from the prediction if the summed score was less than the summed-score cutoff, which was varied from 0 to +40 units to produce the curves shown.

### Comparison to BETAWRAPPRO results

The top hit of BETAWRAPPRO was taken as the prediction for each of the 23 structures; this yielded a set of 276 strands predicted by BETAWRAPPRO. A BETAWRAPPRO strand was taken to be a “correct prediction” if its N-terminal end was within 3 residues of a crystal structure strand as determined by the DSSP analysis found at the PDB website [59].

### Comparison to BETAPRO, PASTA, PSIPRED, JPRED, SALSA, TANGO, and Zyggregator

BETAPRO, PASTA, SALSA, TANGO, PSIPRED, and JPRED were executed using all default settings. Zyggregator was used in fibrillar mode. To avoid bias and in keeping with author suggestions, no additional secondary structure descriptions or alignments were input to BETAPRO or JPRED. To overcome differences in scoring methods, predictions in Table 1 were rated as ‘strong’ (S), ‘weak’ (w), ‘no prediction’ (n), or no data available (x). A prediction was rated as ‘strong’ if more than 2/3 of the strand's length was predicted and if the internal rating system of the program (if present) scored any portion of the strand as greater than 50% of the peak prediction for that sequence. The prediction was rated ‘weak’ if the above conditions were not satisfied, but more than two residues of the strand were predicted at any confidence level. A prediction was indicated as (+) if the requirements for a weak prediction were met, but no separation existed between strand predictions.

## Supporting Information

Table S1 β-helices used in BETASCAN statistical analyses.
(0.04 MB DOC)

Table S2 Leave-one-out analysis of the set of sequences used for analysis by [45],[49]. After clustering by CD-HIT [58] at 40% similarity, a series of partially non-redundant data sets was created, each with one or two cluster(s)' redundant sequences removed as indicated. BETASCAN and PASTA were used to analyze each partially non-redundant data set, and the intersection point of the sensitivity and specificity ROC curves for each algorithm was calculated. Delta indicates the change in score from the full non-redundant data set. Boldface indicates the presence of redundancy in the A-β cluster.
(0.08 MB DOC)

Table S3 Nonredundant set of sequences from aggregative proteins, derived from [45].
(0.15 MB DOC)

## References

[1] Dobson C (2001). The structural basis of protein folding and its links with human
disease.. Philosophical Transactions: Biological Sciences.

[2] Selkoe D (2003). Folding proteins in fatal ways.. Nature.

[3] Dobson C (2003). Protein folding and misfolding.. Nature.

[4] Prusiner SB (2004). Prion Biology and Diseases.

[5] Bucciantini M, Giannoni E, Chiti F, Baroni F, Formigli L (2002). Inherent cytotoxicity of aggregates implies a common origin for
protein misfolding diseases.. Nature.

[6] Fowler DM, Koulov AV, Alory-Jost C, Marks MS, Balch WE (2006). Functional amyloid formation within mammalian tissue.. PLoS biology.

[7] Chapman MR, Robinson LS, Pinkner JS, Roth R, Heuser J (2002). Role of Escherichia coli curli operons in directing amyloid fiber
formation.. Science (New York, NY).

[8] Wickner RB, Edskes HK, Shewmaker F, Nakayashiki T, Laboratory of B (2007). Prions of fungi: inherited structures and biological roles.. Nat Rev Microbiol.

[9] Uptain SM, Lindquist S (2002). Prions as protein-based genetic elements.. Annual review of microbiology.

[10] Si K, Lindquist S, Kandel ER (2003). A neuronal isoform of the aplysia CPEB has prion-like properties.. Cell.

[11] Sunde M, Blake C (1997). The structure of amyloid fibrils by electron microscopy and X-ray
diffraction.. Advances in protein chemistry.

[12] Maddelein ML, Dos Reis S, Duvezin-Caubet S, Coulary-Salin B, Saupe SJ (2002). Amyloid aggregates of the HET-s prion protein are infectious.. Proceedings of the National Academy of Sciences of the United States of
America.

[13] Cascio M, Glazer PA, Wallace BA (1989). The secondary structure of human amyloid deposits as determined
by circular dichroism spectroscopy.. Biochemical and biophysical research communications.

[14] Soto C, Castaäno EM (1996). The conformation of Alzheimer's beta peptide determines
the rate of amyloid formation and its resistance to proteolysis.. The Biochemical journal.

[15] Kajava AV, Squire JM, Parry DA (2006). Beta-structures in fibrous proteins.. Advances in protein chemistry.

[16] Nelson R, Sawaya MR, Balbirnie M, Madsen A, Riekel C (2005). Structure of the cross-beta spine of amyloid-like fibrils.. Nature.

[17] Sawaya MR, Sambashivan S, Nelson R, Ivanova MI, Sievers SA (2007). Atomic structures of amyloid cross-beta spines reveal varied
steric zippers.. Nature.

[18] Petkova AT, Buntkowsky G, Dyda F, Leapman RD, Yau WM (2004). Solid state NMR reveals a pH-dependent antiparallel beta-sheet
registry in fibrils formed by a beta-amyloid peptide.. Journal of molecular biology.

[19] Lèuhrs T, Ritter C, Adrian M, Riek-Loher D, Bohrmann B (2005). 3D structure of Alzheimer's amyloid-beta(1–42)
fibrils.. Proceedings of the National Academy of Sciences of the United States of
America.

[20] Ritter C, Maddelein ML, Siemer AB, Lèuhrs T, Ernst M (2005). Correlation of structural elements and infectivity of the HET-s
prion.. Nature.

[21] Wasmer C, Lange A, Van Melckebeke H, Siemer AB, Riek R (2008). Amyloid fibrils of the HET-s(218–289) prion form a beta
solenoid with a triangular hydrophobic core.. Science.

[22] Wickner R, Dyda F, Tycko R (2008). Amyloid of Rnq1p, the basis of the
[PIN+] prion, has a parallel in-register
{beta}-sheet structure.. Proceedings of the National Academy of Sciences.

[23] Lansbury P (1992). In pursuit of the molecular structure of amyloid plaque: new
technology provides unexpected and critical information.. Biochemistry.

[24] Serpell L (2000). Alzheimer's amyloid fibrils: structure and assembly.. BBA-Molecular Basis of Disease.

[25] Wetzel R (2002). Ideas of Order for Amyloid Fibril Structure.. Structure.

[26] Sipe J, Cohen A (2000). Review: History of the Amyloid Fibril.. Journal of Structural Biology.

[27] DePace AH, Weissman JS (2002). Origins and kinetic consequences of diversity in Sup35 yeast
prion fibers.. Nature structural biology.

[28] Tanaka M, Chien P, Naber N, Cooke R, Weissman J (2004). Conformational variations in an infectious protein determine
prion strain differences.. Nature.

[29] Tessier P, Lindquist S (2007). Prion recognition elements govern nucleation, strain specificity
and species barriers.. Nature.

[30] Krishnan R, Lindquist S (2005). Structural insights into a yeast prion illuminate nucleation and
strain diversity.. Nature.

[31] Chenna R, Sugawara H, Koike T, Lopez R, Gibson TJ (2003). Multiple sequence alignment with the Clustal series of programs.. Nucleic acids research.

[32] Michelitsch M, Weissman J (2000). A census of glutamine/asparagine-rich regions: Implications for
their conserved function and the prediction of novel prions.. Proceedings of the National Academy of Sciences.

[33] Perutz MF, Pope BJ, Owen D, Wanker EE, Scherzinger E (2002). Aggregation of proteins with expanded glutamine and alanine
repeats of the glutamine-rich and asparagine-rich domains of Sup35 and of
the amyloid beta-peptide of amyloid plaques.. Proceedings of the National Academy of Sciences of the United States of
America.

[34] Derkatch IL, Uptain SM, Outeiro TF, Krishnan R, Lindquist SL (2004). Effects of Q/N-rich, polyQ, and non-polyQ amyloids on the de novo
formation of the [PSI+] prion in yeast and
aggregation of Sup35 in vitro.. Proceedings of the National Academy of Sciences of the United States of
America.

[35] Goldfarb LG, Brown P, McCombie WR, Goldgaber D, Swergold GD (1991). Transmissible familial Creutzfeldt-Jakob disease associated with
five, seven, and eight extra octapeptide coding repeats in the PRNP gene.. Proceedings of the National Academy of Sciences of the United States of
America.

[36] DePace AH, Santoso A, Hillner P, Weissman JS (1998). A critical role for amino-terminal glutamine/asparagine repeats
in the formation and propagation of a yeast prion.. Cell.

[37] Cuff JA, Barton GJ (2000). Application of multiple sequence alignment profiles to improve
protein secondary structure prediction.. Proteins.

[38] Rost B, Yachdav G, Liu J (2004). The PredictProtein server.. Nucleic acids research.

[39] Altschul SF, Gish W, Miller W, Myers EW, Lipman DJ (1990). Basic local alignment search tool.. Journal of molecular biology.

[40] Bradley P, Cowen L, Menke M, King J, Berger B (2001). BETAWRAP: successful prediction of parallel beta -helices from
primary sequence reveals an association with many microbial pathogens.. Proceedings of the National Academy of Sciences of the United States of
America.

[41] McDonnell AV, Menke M, Palmer N, King J, Cowen L (2006). Fold recognition and accurate sequence-structure alignment of
sequences directing beta-sheet proteins.. Proteins.

[42] Perutz M, Finch J, Berriman J, Lesk A (2002). Amyloid fibers are water-filled nanotubes.. Proceedings of the National Academy of Sciences.

[43] Wille H, Michelitsch M, Guenebaut V, Supattapone S, Serban A (2002). Structural studies of the scrapie prion protein by electron
crystallography.. Proceedings of the National Academy of Sciences of the United States of
America.

[44] Cheng J, Baldi P (2005). Three-stage prediction of protein beta-sheets by neural networks,
alignments and graph algorithms.. Bioinformatics (Oxford, England).

[45] Fernandez-Escamilla AM, Rousseau F, Schymkowitz J, Serrano L (2004). Prediction of sequence-dependent and mutational effects on the
aggregation of peptides and proteins.. Nat Biotechnol.

[46] Tartaglia GG, Pawar AP, Campioni S, Dobson CM, Chiti F (2008). Prediction of aggregation-prone regions in structured proteins.. J Mol Biol.

[47] Chiti F, Stefani M, Taddei N, Ramponi G, Dobson CM (2003). Rationalization of the effects of mutations on peptide and
protein aggregation rates.. Nature.

[48] Zibaee S, Makin OS, Goedert M, Serpell LC (2007). A simple algorithm locates beta-strands in the amyloid fibril
core of alpha-synuclein, Abeta, and tau using the amino acid sequence alone.. Protein science : a publication of the Protein Society.

[49] Trovato A, Seno F, Tosatto SC (2007). The PASTA server for protein aggregation prediction.. Protein Eng Des Sel.

[50] Trovato A, Chiti F, Maritan A, Seno F (2006). Insight into the structure of amyloid fibrils from the analysis
of globular proteins.. PLoS computational biology.

[51] Heise H, Hoyer W, Becker S, Andronesi OC, Riedel D (2005). Molecular-level secondary structure, polymorphism, and dynamics
of full-length alpha-synuclein fibrils studied by solid-state NMR.. Proceedings of the National Academy of Sciences of the United States of
America.

[52] von Bergen M, Friedhoff P, Biernat J, Heberle J, Mandelkow EM (2000). Assembly of tau protein into Alzheimer paired helical filaments
depends on a local sequence motif ((306)VQIVYK(311)) forming beta structure.. Proceedings of the National Academy of Sciences of the United States of
America.

[53] Kajava AV, Aebi U, Steven AC (2005). The parallel superpleated beta-structure as a model for amyloid
fibrils of human amylin.. Journal of molecular biology.

[54] Sachse C, Fandrich M, Grigorieff N (2008). Paired beta-sheet structure of an Abeta(1–40) amyloid
fibril revealed by electron microscopy.. Proc Natl Acad Sci U S A.

[55] Jenkins J, Pickersgill R (2001). The architecture of parallel ß-helices and related
folds.. Progress in Biophysics and Molecular Biology.

[56] Esposito G, Viglino P, Novak M, Cattaneo A (2000). The solution structure of the C-terminal segment of tau protein.. Journal of peptide science : an official publication of the European
Peptide Society.

[57] McGuffin LJ, Bryson K, Jones DT (2000). The PSIPRED protein structure prediction server.. Bioinformatics.

[58] Li W, Godzik A (2006). Cd-hit: a fast program for clustering and comparing large sets of
protein or nucleotide sequences.. Bioinformatics.

[59] Berman HM, Westbrook J, Feng Z, Gilliland G, Bhat TN (2000). The Protein Data Bank.. Nucleic acids research.

[60] Frishman D, Argos P (1995). Knowledge-based protein secondary structure assignment.. Proteins.

[61] Penel S, Morrison RG, Dobson PD, Mortishire-Smith RJ, Doig AJ (2003). Length preferences and periodicity in beta-strands. Antiparallel
edge beta-sheets are more likely to finish in non-hydrogen bonded rings.. Protein engineering.

[62] Kabsch W, Sander C (1983). Dictionary of protein secondary structure: pattern recognition of
hydrogen-bonded and geometrical features.. Biopolymers.

