# Supplementary material for: BETASCAN: Probable β-amyloids Identified by Pairwise Probabilistic Analysis
Source: PLoS Comput Biol. 2009 Mar 27;5(3):e1000333. doi: 10.1371/journal.pcbi.1000333 (PMC2653728; doi:10.1371/journal.pcbi.1000333)
Supplement: Table S1 — β-helices used in BETASCAN statistical analyses. (0.04 MB DOC) [file pcbi.1000333.s001.doc]

Table S1, β-helices used in BETASCAN statistical analyses.

| PDB id | Protein | Species |
| --- | --- | --- |
| 1air | Pectate Lyase E | Erwinia chrysanthemi |
| 1bhe | Polygalacturonase | Erwinia carotovora ssp. Carotovora |
| 1bn8 | Pectate Lyase | Bacillus subtilis |
| 1czf | Endo-Polygalacturonase II | Aspergillus niger |
| 1dab | Virulence Factor P.69 Pertactin | Bordetella pertussis |
| 1dbg | Chondroitinase B | Flavobacterium heparinum |
| 1ee6 | Pectate Lyase | Bacillus sp. Strain KSM-p15. |
| 1h80 | Iota-Carrageenase Of | Alteromonas fortis |
| 1hg8 | Endopolygalacturonase | Fusarium moniliforme |
| 1ib4 | Polygalacturonase | Aspergillus aculeatus |
| 1idj | Pectin Lyase A | Aspergillus sp. |
| 1jrg | Pectate Lyase A | Erwinia chrysanthemi |
| 1jta | Pectate Lyase A (C2 Form) | Erwinia chrysanthemi |
| 1k5c | Endopolygalacturonase I | Stereum purpureum |
| 1ktw | Iota-Carrageenase | Alteromonas sp. Atcc43554 |
| 1nhc | Endopolygalacturonase I | Aspergillus niger |
| 1ogm | Dex49a From | Penicillium minioluteum |
| 1qcx | Pectin Lyase B | Aspergillus niger |
| 1qjv | Pectin Methylesterase PEMA | Erwinia chrysanthemi |
| 1rmg | Rhamnogalacturonase A | Aspergillus aculeatus |
| 1rwr | Filamentous Hemagglutinin Secretion Domain | Bordetella pertussis |
| 1tsp | Tailspike Protein | Bacteriophage P22 |
| 2pec | Pectate Lyase C | Erwinia chrysanthemi |
