# Supplementary material for: BETASCAN: Probable β-amyloids Identified by Pairwise Probabilistic Analysis
Source: PLoS Comput Biol. 2009 Mar 27;5(3):e1000333. doi: 10.1371/journal.pcbi.1000333 (PMC2653728; doi:10.1371/journal.pcbi.1000333)
Supplement: Table S2 — Leave-one-out analysis of the set of sequences used for analysis by [45],[49]. After clustering by CD-HIT [58] at 40% similarity, a series of partially non-redundant data sets was created, each with one or two cluster(s)' redundant sequences removed as indicated. BETASCAN and PASTA were used to analyze each partially non-redundant data set, and the intersection point of the sensitivity and specificity ROC curves for each algorithm was calculated. Delta indicates the change in score from the full non-redundant data set. Boldface indicates the presence of redundancy in the A-β cluster. (0.08 MB DOC) [file pcbi.1000333.s002.doc]

Table S2. Leave-one-out analysis of the set of sequences used for analysis by [45, 49]. After clustering by CD-HIT [58] at 40% similarity, a series of partially non-redundant data sets was created, each with one or two cluster(s)’ redundant sequences removed as indicated. BETASCAN and PASTA were used to analyze each partially non-redundant data set, and the intersection point of the sensitivity and specificity ROC curves for each algorithm was calculated. Delta indicates the change in score from the full non-redundant data set. Boldface indicates the presence of redundancy in the A-beta cluster.

|  | | | | | |  | |  |
| --- | --- | --- | --- | --- | --- | --- | --- | --- |
| **Data set** | **Sequences** | | **BETASCAN ROC intersection** | | **PASTA ROC intersection** | | | |
|  | Included | Excluded |  | Delta |  | | Delta | |
| *Full NR set* | *122* | *56* | *0.807* | *0* | *0.8* | | *0* | |
| Original TANGO/PASTA | 178 | 0 | 0.722 | **-0.085** | 0.807 | | 0.007 | |
| A-beta NR | 154 | 24 | 0.797 | -0.01 | 0.802 | | 0.002 | |
| Ada NR | 168 | 10 | 0.727 | **-0.08** | 0.808 | | 0.008 | |
| Alpha-synuclein NR | 175 | 3 | 0.74 | **-0.067** | 0.812 | | 0.012 | |
| Beta-microglobulin NR | 176 | 2 | 0.722 | **-0.085** | 0.813 | | 0.013 | |
| BPTI NR | 175 | 3 | 0.716 | **-0.091** | 0.802 | | 0.002 | |
| myohemerithin NR | 176 | 2 | 0.723 | **-0.084** | 0.804 | | 0.004 | |
| plastocyanin NR | 171 | 7 | 0.713 | **-0.094** | 0.806 | | 0.006 | |
| sprectrin NR | 176 | 2 | 0.724 | **-0.083** | 0.822 | | 0.022 | |
| t-Protein NR | 168 | 10 | 0.712 | **-0.095** | 0.806 | | 0.006 | |
| A-beta + Ada NR | 152 | 26 | 0.802 | -0.005 | 0.802 | | 0.002 | |
| A-beta + Alpha-synuclein NR | 151 | 27 | 0.819 | 0.012 | 0.812 | | 0.012 | |
| A-beta + Beta-microglobulin NR | 151 | 27 | 0.797 | -0.01 | 0.807 | | 0.007 | |
| A-beta + BPTI NR | 152 | 26 | 0.792 | -0.015 | 0.797 | | -0.003 | |
| A-beta + myohemerithin NR | 151 | 27 | 0.794 | -0.013 | 0.799 | | -0.001 | |
| A-beta + plastocyanin NR | 147 | 31 | 0.792 | -0.015 | 0.799 | | -0.001 | |
| A-beta + spectrin NR | 152 | 26 | 0.799 | -0.008 | 0.804 | | 0.004 | |
| A-beta + t-Protein NR | 144 | 34 | 0.802 | -0.005 | 0.786 | | -0.014 | |
