# Supplementary material for: BETASCAN: Probable β-amyloids Identified by Pairwise Probabilistic Analysis
Source: PLoS Comput Biol. 2009 Mar 27;5(3):e1000333. doi: 10.1371/journal.pcbi.1000333 (PMC2653728; doi:10.1371/journal.pcbi.1000333)
Supplement: Table S3 — Nonredundant set of sequences from aggregative proteins, derived from [45]. (0.15 MB DOC) [file pcbi.1000333.s003.doc]

Table S3. Nonredundant set of sequences from aggregative proteins, derived from [45].

| 1Cro | MQTLSERLKKRRIALKY |
| --- | --- |
| 2Cro | YKMTQTELATKAGVK |
| 3Cro | YKQQSIQLIEAGVTKR |
| 4Cro | TKRPRFLYEIAMALNSD |
| 5Cro | AMALNCDPVWLQYGTKRGKA |
| Acyl-phosphatase17-Jan | STAQSLKSVDYEVFGRV |
| Acyl-phosphatase18-33 | QGVSFRMYTEDEARKI |
| Acyl-phosphatase34-53 | GVVGWVKNTSKGTVTGQVQG |
| Acyl-phosphatase54-68 | PEDKVNSMKSWLSKV |
| Acyl-phosphatase69-85 | GSPSSRIDRTNFSNEKT |
| Acyl-phosphatase86-98 | ISKLEYSNFSVRY |
| Ada-2HH1-Wt | VPSNEEQIKNLLQLEAQEHLQY |
| Ada-2HH2-WT | FVNVQAVKVFLESQGIAY |
| Alpha-synucleinNAC1-18 | EQVTNVGGAVVTGVTAVA |
| Alpha-synucleinNAC1-18s | TVNGVGEVTATAVQGVAV |
| Alpha-synucleinNAC6-14 | VGGAVVTGV |
| Amyloid-betaAB3 | HQKLVFFAE |
| Amyloid-betaHABP5 | KKPVFFAED |
| Amyloid-betaWhole | DAEFRHDSGYEVHHQKLVFFAEDVGSNKGAIIGLMVGGVV |
| AraAra1 | AVGKSNLLSRYARNEFSA |
| AraAra2 | RFRAVTSAYYRGAVG |
| AraAra3 | TRRTTFESVGRWLDELKIHSD |
| AraAra4 | AVSVEEGKALAEEEGLF |
| AraAra5 | STNVKTAFEMVILDIYNNV |
| Beta-microglobulinA | IQRTPKIQVYSRHPAE |
| Beta-microglobulinB | NGKSNFLNCYVSG |
| Beta-microglobulinC | FHPSDIEVDLLK |
| Beta-microglobulinD | NGERIEKVEHSDLSFSKD |
| Beta-microglobulinE1 | DWSFYLLYYTEFTPTGKDEYA |
| Beta-microglobulinF | PTGKDEYACRVNHVT |
| BPTIP1-15 | RPDFSLEPPYTGPSK |
| BPTIP16-28 | ARIIRYFYNAKAG |
| BPTIP29-44 | LSQTFVYGGSRAKRNN |
| BPTIP45-58 | 0FKSAEDSMRTSGGA |
| CheYCheY1 | DFSTMRRIVRNLLKELGYN |
| CheYCheY2 | EDGVDALNKLQAGGY |
| CheYCheY3 | MDGLELLKTIRADSAY |
| CheYCheY4 | AKKENIIAAAQAGASGY |
| CheYCheY5 | PFTAATLEEKLNKIFEKLGMY |
| ComComA1 | DHPAVMEGTKTILETDSNLS |
| ComComA2 | EPSEQFIKQHDFSSY |
| ComComA3 | VNGMELSKQILQENPH |
| ComComA4 | EVEDYFEEAIRAGLH |
| ComComA5 | TESKEKITQYIYHVLNGEIL |
| FlaxodoxinFXN1 | GTGNTEKMAELIAKGIIESGKDY |
| FlaxodoxinFXN3 | EESEFEPFIEEISTKISY |
| FlaxodoxinFXN4 | GDGKWMRDFEQRMNGYGSV |
| FlaxodoxinFXN5 | EPDEAEQDSIEFGKKIANIY |
| GlutexAlpha-4 | DQKEAALVDMVNDGVEDLRCKYATLIYT |
| GlutexAlpha-5 | YEAGKEKYVKELPEHLKPFETLLSQ |
| GlutexAlpha-6 | QISFADYNLLDLLRIHQVLN |
| GlutexAlpha-7 | PLLSAYVARLSA |
| GlutexAlpha-8 | PKIKAFLA |
| myoglobinAB-Domain | VLSEGEWQLVLHVWAKVEA |
| myoglobinA-Helix | EGEWQLVLHVWAKVEADVAGHGQDILIRLFK |
| myoglobinBC-Turn | KSHPET |
| myoglobinB-Helix | DVAGHGQDILIRLFKS |
| myoglobinCCD-Domai | HPETLEKFDRFKHLK |
| myoglobinD-Helix | TEAEMKA |
| myoglobinEF-Turn | SEDLKKHGVTVLTALGAILK |
| myoglobinE-Helix | KKGHHEAE |
| myoglobinFG-Turn | ATKHKIP |
| myoglobinF-helix | ELKPLAQSHA |
| MyohemerithinAB-loop | Am-YEQLDEEHKKIFKGIFDCIRD |
| MyohemerithinA-helix | RDNSA |
| MyohemerithinBC-loop | DAAKYSEV |
| MyohemerithinB-helix | SAPNLATLVKVTTNHFTHEEAMMD |
| MyohemerithinCD-loop | GLSAPVD |
| MyohemerithinC-helix | EVVPHKKMHKDFLEKIGGL |
| MyohemerithinC-terminal | GTDFKYKGKL |
| MyohemerithinD-helix | AKNVDYCKEWLVNHIK |
| MyohemerithinN-terminal | GWEIPEPYVWDESFRVFY |
| PlastocyaninPc-1 | LEVLLGSG |
| PlastocyaninPc-10 | IPAGVDAVKISM |
| PlastocyaninPc-10a | EIPAGV |
| PlastocyaninPc-10b | DAVKIS |
| PlastocyaninPc-11 | MPEEELL |
| PlastocyaninPc-12 | MPEEELLNAPGETYVVTL |
| PlastocyaninPc-13b | APGET |
| PlastocyaninPc-14 | GETYVVTL |
| PlastocyaninPc-14a | ETYVVT |
| PlastocyaninPc-15 | VTLDTKGTY |
| PlastocyaninPc-16 | GTYSFYT |
| PlastocyaninPc-16a | TYSFYC |
| PlastocyaninPc-18 | MVGKVTVN |
| PlastocyaninPc-19 | GTVSFVTSPHQGAGMVGKVTVN |
| PlastocyaninPc-2 | LEVLLGSGDGSLVFV |
| PlastocyaninPc-3 | SLVFVPSEFS |
| PlastocyaninPc-5 | SEFSVPSGEK |
| PlastocyaninPc-6 | KIVFKNNA |
| PlastocyaninPc-6a | GEKIVFKNNAGFPHNVVFDE |
| PlastocyaninPc-8 | KNNAGFPHNV |
| PL-B1PL-B1-114-138-pH-2.4 | KGTFEKATSEAYAYADTLKKDNGEY |
| PL-B1PL-B1-136-155D-pH-6.1 | GEYTVDVADKGYTLNIKFAGD |
| PL-B1PL-B1-95-114-pH-4.1 | VTIKANLIFANGFTQTAEFKG |
| ProteinGProteinG21-40 | TYKLINGKTLKGETTTEA |
| ProteinGProteinG2-19 | GDAATAEKVFKQYANDNGVD |
| ProteinGProteinG41-56 | GEWTYDDATKTFTVTE |
| RasP21A | GVGKSALTIQLIQNHFVY |
| RasP21B | EYSAMRDQYMRTGEG |
| RasP21C | INNTKSFEDIHQYREQIKRVKDS |
| RasP21D | ARTVESRQAQDLARSYGIP |
| RasP21E | RQGVEDAFYTLVREIRQHK |
| Ribosome-L9Alpha-1 | GYANNFLFKQG |
| Ribosome-L9Alpha-2 | TPANLKALEAQKQKEQR |
| Ribosome-L9Beta-1 | MKVIFLKDVKG |
| Ribosome-L9Beta-2 | KGKKGEIKNVAD |
| Ribosome-L9Beta-3 | LAIEATPA |
| Spectrin-SH3M-2 | AYVKKLDSGTGKELVLAL |
| Spectrin-SH3M-4 | YDYQEKSPREVTMKKGD |
| Spectrin-SH3M-68 | DILTLLNSTNKDWWKVEVNDRQGFVPA |
| Spectrin-SH3M-C | GGKDWWKVGG |
| t-ProteinK19 | PGGGKVQIVYKPV |
| t-ProteinK19d | PGGGKVYKPV |
| t-ProteinK19Gluc4 | QTAPVPMPDLKNVKSKIGSTE |
| t-ProteinK19Gluc41 | NLKHQPGGGKVQIVYKPVDLSKVTSKCGSLGNIHHKPGGGQVE |
| t-ProteinK19Gluc42 | VKSE |
| t-ProteinK19Gluc78 | QTAPVPMPD |
| t-ProteinV313-K321 | VDLSKVTSK |
| t-ProteinV335-E342 | GQVEVSKE |
